# Poly[μ_4_-glutarato-di-μ_3_-glutarato-bis­(1,10-phenanthroline)diyttrium(III)]

**DOI:** 10.1107/S1600536811027188

**Published:** 2011-07-13

**Authors:** Ling Jin, Hong-lin Zhu

**Affiliations:** a Center of Applied Solid State Chemistry Research, Ningbo University, Ningbo, Zhejiang 315211, People’s Republic of China

## Abstract

In the title complex, [Y_2_(C_5_H_6_O_4_)_3_(C_12_H_8_N_2_)_2_]_*n*_, three glutarate groups and two 1,10-phenanthroline mol­ecules surround the two Y^III^ ions. Both Y^III^ ions are coordinated by two N atoms from the 1,10-phenanthroline, seven O atoms from five glutarate groups in a distorted tricapped trigonal–prismatic geometry. The Y^III^ ions are bridged by glutarate ligands in three modes, forming a layered, polymeric structure. The resulting layers are assembled by π–π stacking inter­actions [centroid–centroid distances = 3.740 (3) and 3.571 (3) Å] into a three-dimensional supra­molecular architecture.

## Related literature

For general background to applications of coordination polymers as functional materials, see: Koo *et al.* (2010 ▶). For related structures, see: Zhang *et al.* (2003 ▶): Yin & Yu (2007 ▶).
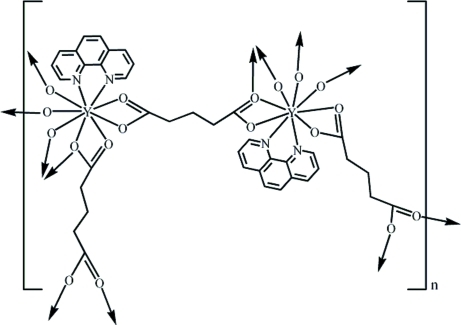


## Experimental

### 

#### Crystal data

[Y_2_(C_5_H_6_O_4_)_3_(C_12_H_8_N_2_)_2_]
*M* *_r_* = 928.52
Triclinic, 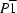

*a* = 8.7681 (18) Å
*b* = 13.418 (3) Å
*c* = 16.410 (3) Å
α = 83.83 (3)°
β = 84.41 (3)°
γ = 75.09 (3)°
*V* = 1849.9 (6) Å^3^
*Z* = 2
Mo *K*α radiation
μ = 3.19 mm^−1^
*T* = 293 K
0.23 × 0.17 × 0.08 mm

#### Data collection

Rigaku R-AXIS RAPID diffractometer
Absorption correction: multi-scan (*ABSCOR*; Higashi, 1995 ▶) *T*_min_ = 0.790, *T*_max_ = 0.810
17986 measured reflections
8240 independent reflections
5620 reflections with *I* > 2σ(*I*)
*R*_int_ = 0.056

#### Refinement

*R*[*F* ^2^ > 2σ(*F* ^2^)] = 0.045
*wR*(*F* ^2^) = 0.076
*S* = 1.02
8240 reflections
514 parameters
H-atom parameters constrained
Δρ_max_ = 0.50 e Å^−3^
Δρ_min_ = −0.43 e Å^−3^

### 

Data collection: *RAPID-AUTO* (Rigaku, 1998 ▶); cell refinement: *RAPID-AUTO*; data reduction: *CrystalStructure* (Rigaku/MSC, 2004 ▶); program(s) used to solve structure: *SHELXS97* (Sheldrick, 2008 ▶); program(s) used to refine structure: *SHELXL97* (Sheldrick, 2008 ▶); molecular graphics: *ORTEPII* (Johnson, 1976) ▶; software used to prepare material for publication: *SHELXL97*.

## Supplementary Material

Crystal structure: contains datablock(s) global, I. DOI: 10.1107/S1600536811027188/kp2336sup1.cif

Structure factors: contains datablock(s) I. DOI: 10.1107/S1600536811027188/kp2336Isup2.hkl

Additional supplementary materials:  crystallographic information; 3D view; checkCIF report

## Figures and Tables

**Table 1:** Selected bond lengths (Å)

Y1—O2^i^	2.314 (2)
Y1—O1^ii^	2.314 (2)
Y1—O6^iii^	2.315 (2)
Y1—O3	2.386 (2)
Y1—O5	2.441 (3)
Y1—O4	2.455 (3)
Y1—O6	2.537 (2)
Y1—N2	2.538 (3)
Y1—N1	2.592 (3)
Y2—O10^iv^	2.269 (3)
Y2—O12^v^	2.317 (2)
Y2—O11^i^	2.329 (2)
Y2—O7	2.375 (3)
Y2—O9	2.397 (3)
Y2—O8	2.413 (3)
Y2—N4	2.570 (3)
Y2—N3	2.649 (3)
Y2—O10	2.823 (3)

Symmetry codes: (i) 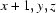; (ii) 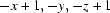; (iii) 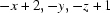; (iv) 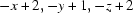; (v) 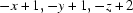.

**Fig. 1.**
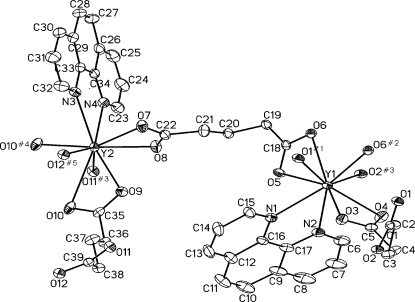
ORTEP view of the title compound. The dispalcement ellipsoids are drawn at the 30% probability level. [Symmetry codes used: (#1)-x + 1, -y, -z + 1;(#2)-x + 2, -y, -z + 1; (#3)x + 1, y, z;(#4)-x + 2, -y + 1, -z + 2;(#5)-x + 1, -y + 1, -z + 2].

**Fig. 2.**
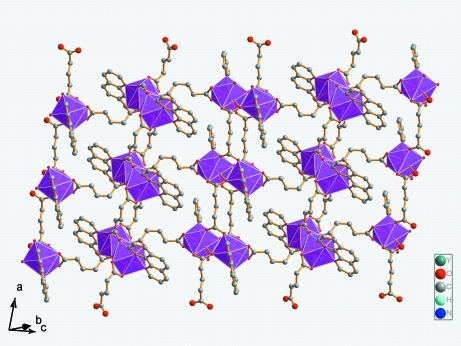
The view of layered structure parallel to (011).

## supplementary crystallographic information

### Comment

In the past decades, growing attention has been paid to rational design and synthesis of coordination polymers due to their potential applications as functional materials (Koo *et al.*, 2010). We report here the preparation and crystal structures of one interesting coordination polymers constructed by Yttrium(III) centers, 1,10-phenanthroline and glutarate, namely [Y_2_(phen)_2_(glu)_3_]_n_.

The asymmetric unit of the title compound contains two Y^III^ ions, two phen molecules and three glutarate anions. Both Y^III^ ions are coordinated by seven oxygen atoms from five glutarate ligands and two nitrogen atoms from a chelating phen ligand (Fig. 1). The Y-O/N bond distances fall in a range from 2.269 to 2.823 Å (Table 1). Each of Y^III^ ions exhibits the coordination geometry of distorted tricapped trigonal prism. The glutarate ligands exhibit three types of linking modes to bridge Y^III^ ions into the polymeric structure: (*a*) bridging bidentate and chelating bidentate; (*b*) chelating/bridging tridentate and bridging bidentate; (*c*) chelating/bridging tridentate and chelating bidentate (Zhang *et al.*, 2003). The Y^III^ ions are bridged by glutarate anions to form layers parallel to (011) (Fig. 2). π–π stacking interactions of phen ligands with separation distances between centres of gravity of 3.740 (3)Å and 3.571 (3) Å [involving the rings: N1-C15-C14_C13_C12-C16 and C9-C10-C11-C12-C16-C17 with symmetry operation of the second ring (1-x,1-y,1-z), and the ring C26-C27-C28-C29-C33-C34 and its symmetry related one (3-x,-y,2-z)] generate a three-dimensional structure (Yin *et al.*, 2007).

### Experimental

YCl_3_.nH_2_O were prepared by dissolving 0.0339 g Y_2_O_3_ (0.15 mmol) in dilute hydrochloric acid and then dried. A mixture of YCl_3_.nH_2_O, H_2_glu (0.0396 g, 0.30 mmol), phen (0.0595 g, 0.30 mmol) and H_2_O (10 mL) was stirred and adjusted to pH 4.0 with a 1*M* NaOH solution, then transferred and sealed into an 23 mL-Teflon-lined autoclave, which was heated at 443 K for three days. After cooling to room temperature at a rate of 10 K /3 h, colourless block-like crystals were obtained, washed with ethanol and dried in air.

### Refinement

H atoms bonded to C atoms were palced in geometrically calculated position and were refined using a riding model, with *U*_iso_(H) = 1.2*U*_eq_(C). H atoms attached to O atoms were found in a difference Fourier synthesis and were refined using a riding model, with the O–H distances fixed as initially found and with *U*_iso_(H) values set at 1.2 *U*eq(O).

### Crystal data

**Table:**

[Y_2_(C_5_H_6_O_4_)_3_(C_12_H_8_N_2_)_2_]	*Z* = 2
*M**_r_* = 928.52	*F*(000) = 940
Triclinic, *P*1	*D*_x_ = 1.667 Mg m^−^^3^
Hall symbol: -P 1	Mo *K*α radiation, λ = 0.71073 Å
*a* = 8.7681 (18) Å	Cell parameters from 17986 reflections
*b* = 13.418 (3) Å	θ = 3.1–27.5°
*c* = 16.410 (3) Å	µ = 3.19 mm^−^^1^
α = 83.83 (3)°	*T* = 293 K
β = 84.41 (3)°	Block, colorless
γ = 75.09 (3)°	0.23 × 0.17 × 0.08 mm
*V* = 1849.9 (6) Å^3^	

### Data collection

**Table:**

Rigaku R-AXIS RAPID diffractometer	8240 independent reflections
Radiation source: fine-focus sealed tube	5620 reflections with *I* > 2σ(*I*)
graphite	*R*_int_ = 0.056
Detector resolution: 0 pixels mm^-1^	θ_max_ = 27.5°, θ_min_ = 3.1°
ω scans	*h* = −10→11
Absorption correction: multi-scan (*ABSCOR*; Higashi, 1995)	*k* = −15→17
*T*_min_ = 0.790, *T*_max_ = 0.810	*l* = −21→21
17986 measured reflections	

### Refinement

**Table:**

Refinement on *F*^2^	Primary atom site location: structure-invariant direct methods
Least-squares matrix: full	Secondary atom site location: difference Fourier map
*R*[*F*^2^ > 2σ(*F*^2^)] = 0.045	Hydrogen site location: inferred from neighbouring sites
*wR*(*F*^2^) = 0.076	H-atom parameters constrained
*S* = 1.02	*w* = 1/[σ^2^(*F*_o_^2^) + (0.0145*P*)^2^ + 1.8882*P*] where *P* = (*F*_o_^2^ + 2*F*_c_^2^)/3
8240 reflections	(Δ/σ)_max_ = 0.001
514 parameters	Δρ_max_ = 0.50 e Å^−^^3^
0 restraints	Δρ_min_ = −0.43 e Å^−^^3^

### Special details

**Table:**

Geometry. All e.s.d.'s (except the e.s.d. in the dihedral angle between two l.s. planes) are estimated using the full covariance matrix. The cell e.s.d.'s are taken into account individually in the estimation of e.s.d.'s in distances, angles and torsion angles; correlations between e.s.d.'s in cell parameters are only used when they are defined by crystal symmetry. An approximate (isotropic) treatment of cell e.s.d.'s is used for estimating e.s.d.'s involving l.s. planes.
Refinement. Refinement of *F*^2^ against ALL reflections. The weighted *R*-factor *wR* and goodness of fit *S* are based on *F*^2^, conventional *R*-factors *R* are based on *F*, with *F* set to zero for negative *F*^2^. The threshold expression of *F*^2^ > σ(*F*^2^) is used only for calculating *R*-factors(gt) *etc*. and is not relevant to the choice of reflections for refinement. *R*-factors based on *F*^2^ are statistically about twice as large as those based on *F*, and *R*- factors based on ALL data will be even larger.

### Fractional atomic coordinates and isotropic or equivalent isotropic displacement parameters (Å^2^)

**Table:**

	*x*	*y*	*z*	*U*_iso_*/*U*_eq_	
Y1	0.86495 (3)	0.13918 (2)	0.47569 (2)	0.02109 (9)	
Y2	1.08369 (4)	0.36263 (3)	0.95322 (2)	0.02321 (9)	
O1	0.2367 (3)	−0.05407 (18)	0.40997 (15)	0.0315 (6)	
O2	0.0995 (2)	0.11228 (18)	0.39369 (15)	0.0289 (6)	
C1	0.2239 (4)	0.0409 (3)	0.3887 (2)	0.0235 (8)	
C2	0.3754 (4)	0.0700 (3)	0.3574 (2)	0.0288 (8)	
H2A	0.4246	0.0274	0.3132	0.035*	
H2B	0.4465	0.0527	0.4014	0.035*	
C3	0.3610 (4)	0.1817 (3)	0.3264 (2)	0.0301 (9)	
H3A	0.3145	0.2255	0.3703	0.036*	
H3B	0.2910	0.2002	0.2819	0.036*	
C4	0.5215 (4)	0.2014 (3)	0.2958 (2)	0.0352 (9)	
H4A	0.5637	0.1603	0.2498	0.042*	
H4B	0.5054	0.2736	0.2754	0.042*	
C5	0.6438 (4)	0.1773 (3)	0.3585 (2)	0.0289 (8)	
O3	0.6014 (3)	0.1760 (2)	0.43423 (16)	0.0344 (6)	
O4	0.7872 (3)	0.1615 (2)	0.33384 (16)	0.0381 (7)	
C6	0.9464 (5)	0.3403 (3)	0.3483 (3)	0.0496 (12)	
H6A	0.9873	0.2855	0.3158	0.059*	
C7	0.9710 (5)	0.4368 (4)	0.3198 (3)	0.0716 (18)	
H7A	1.0257	0.4459	0.2692	0.086*	
C8	0.9139 (5)	0.5172 (4)	0.3667 (4)	0.0727 (18)	
H8A	0.9322	0.5816	0.3492	0.087*	
C9	0.8272 (5)	0.5034 (3)	0.4419 (3)	0.0506 (12)	
C10	0.7514 (6)	0.5851 (3)	0.4922 (4)	0.0662 (15)	
H10A	0.7674	0.6508	0.4780	0.079*	
C11	0.6573 (6)	0.5693 (3)	0.5594 (3)	0.0636 (15)	
H11A	0.6075	0.6245	0.5901	0.076*	
C12	0.6328 (5)	0.4693 (3)	0.5841 (3)	0.0435 (10)	
C13	0.5314 (5)	0.4493 (3)	0.6519 (3)	0.0529 (12)	
H13A	0.4770	0.5028	0.6833	0.063*	
C14	0.5121 (5)	0.3519 (3)	0.6718 (3)	0.0510 (12)	
H14A	0.4435	0.3385	0.7160	0.061*	
C15	0.5979 (4)	0.2725 (3)	0.6243 (2)	0.0381 (10)	
H15A	0.5863	0.2058	0.6391	0.046*	
C16	0.7104 (4)	0.3848 (3)	0.5381 (2)	0.0303 (9)	
C17	0.8068 (4)	0.4031 (3)	0.4656 (3)	0.0352 (9)	
N1	0.6947 (3)	0.2871 (2)	0.55945 (19)	0.0301 (7)	
N2	0.8689 (3)	0.3220 (2)	0.41867 (19)	0.0334 (8)	
O5	1.0269 (3)	0.19778 (18)	0.56407 (17)	0.0349 (6)	
O6	1.0951 (3)	0.03082 (18)	0.55413 (15)	0.0283 (6)	
C18	1.1170 (4)	0.1111 (3)	0.5808 (2)	0.0253 (8)	
C19	1.2573 (4)	0.0989 (3)	0.6293 (2)	0.0327 (9)	
H19A	1.3475	0.1038	0.5913	0.039*	
H19B	1.2809	0.0300	0.6575	0.039*	
C20	1.2400 (4)	0.1761 (3)	0.6919 (2)	0.0302 (8)	
H20A	1.2121	0.2453	0.6645	0.036*	
H20B	1.3413	0.1667	0.7146	0.036*	
C21	1.1166 (4)	0.1671 (3)	0.7617 (2)	0.0391 (10)	
H21A	1.0130	0.1869	0.7400	0.047*	
H21B	1.1351	0.0953	0.7836	0.047*	
C22	1.1154 (4)	0.2328 (3)	0.8310 (2)	0.0303 (8)	
O7	1.0446 (3)	0.2144 (2)	0.89935 (16)	0.0391 (7)	
O8	1.1848 (3)	0.30470 (19)	0.81974 (16)	0.0350 (6)	
N3	1.1495 (3)	0.2053 (2)	1.06709 (19)	0.0322 (7)	
N4	1.3751 (3)	0.2628 (2)	0.96125 (19)	0.0309 (7)	
C23	1.4860 (4)	0.2898 (3)	0.9096 (3)	0.0397 (10)	
H23A	1.4543	0.3431	0.8689	0.048*	
C24	1.6475 (4)	0.2434 (3)	0.9121 (3)	0.0484 (12)	
H24A	1.7211	0.2678	0.8762	0.058*	
C25	1.6946 (4)	0.1624 (3)	0.9681 (3)	0.0482 (12)	
H25A	1.8018	0.1304	0.9706	0.058*	
C26	1.5831 (4)	0.1261 (3)	1.0222 (3)	0.0399 (10)	
C27	1.6204 (5)	0.0371 (3)	1.0784 (3)	0.0488 (12)	
H27A	1.7254	−0.0002	1.0809	0.059*	
C28	1.5091 (5)	0.0050 (3)	1.1281 (3)	0.0505 (12)	
H28A	1.5382	−0.0539	1.1641	0.061*	
C29	1.3447 (5)	0.0601 (3)	1.1268 (3)	0.0393 (10)	
C30	1.2247 (5)	0.0300 (3)	1.1789 (3)	0.0477 (11)	
H30A	1.2485	−0.0291	1.2152	0.057*	
C31	1.0733 (5)	0.0883 (3)	1.1757 (3)	0.0517 (12)	
H31A	0.9926	0.0709	1.2109	0.062*	
C32	1.0410 (5)	0.1744 (3)	1.1188 (3)	0.0453 (11)	
H32A	0.9366	0.2129	1.1169	0.054*	
C33	1.3023 (4)	0.1483 (3)	1.0723 (2)	0.0310 (9)	
C34	1.4212 (4)	0.1816 (3)	1.0173 (2)	0.0316 (9)	
O9	0.8805 (3)	0.4364 (2)	0.86084 (17)	0.0397 (7)	
O10	0.8741 (3)	0.5612 (2)	0.93698 (16)	0.0369 (6)	
C35	0.8169 (4)	0.5247 (3)	0.8827 (2)	0.0267 (8)	
C36	0.6670 (4)	0.5873 (3)	0.8467 (2)	0.0294 (8)	
H36A	0.6680	0.5733	0.7899	0.035*	
H36B	0.6593	0.6605	0.8482	0.035*	
C37	0.5252 (4)	0.5590 (3)	0.8961 (2)	0.0366 (10)	
H37A	0.5392	0.4847	0.8975	0.044*	
H37B	0.5243	0.5758	0.9522	0.044*	
C38	0.3649 (4)	0.6125 (3)	0.8636 (2)	0.0299 (9)	
H38A	0.3441	0.6865	0.8675	0.036*	
H38B	0.3668	0.6009	0.8062	0.036*	
C39	0.2333 (4)	0.5717 (3)	0.9121 (2)	0.0254 (8)	
O11	0.2193 (3)	0.48505 (19)	0.89713 (16)	0.0320 (6)	
O12	0.1485 (3)	0.62867 (18)	0.96418 (15)	0.0301 (6)	

### Atomic displacement parameters (Å^2^)

**Table:**

	*U*^11^	*U*^22^	*U*^33^	*U*^12^	*U*^13^	*U*^23^
Y1	0.01882 (17)	0.01929 (18)	0.0244 (2)	−0.00400 (12)	−0.00107 (13)	−0.00074 (15)
Y2	0.01906 (17)	0.0280 (2)	0.0232 (2)	−0.00726 (13)	0.00089 (14)	−0.00379 (16)
O1	0.0322 (13)	0.0228 (14)	0.0346 (16)	−0.0032 (10)	0.0066 (11)	0.0014 (11)
O2	0.0202 (12)	0.0280 (14)	0.0339 (15)	−0.0022 (10)	0.0023 (10)	0.0042 (11)
C1	0.0223 (17)	0.025 (2)	0.022 (2)	−0.0055 (13)	−0.0008 (14)	−0.0014 (15)
C2	0.0203 (17)	0.031 (2)	0.033 (2)	−0.0043 (14)	−0.0017 (15)	−0.0011 (17)
C3	0.0236 (18)	0.030 (2)	0.039 (2)	−0.0097 (14)	−0.0111 (15)	0.0052 (17)
C4	0.032 (2)	0.043 (2)	0.034 (2)	−0.0173 (16)	−0.0100 (16)	0.0111 (19)
C5	0.029 (2)	0.022 (2)	0.034 (2)	−0.0070 (14)	−0.0031 (16)	0.0049 (16)
O3	0.0246 (13)	0.0450 (17)	0.0326 (16)	−0.0065 (11)	−0.0026 (11)	−0.0040 (13)
O4	0.0224 (13)	0.0559 (18)	0.0341 (16)	−0.0092 (11)	−0.0009 (11)	0.0019 (13)
C6	0.046 (2)	0.040 (3)	0.051 (3)	−0.0022 (19)	0.013 (2)	0.014 (2)
C7	0.063 (3)	0.047 (3)	0.080 (4)	0.004 (2)	0.030 (3)	0.028 (3)
C8	0.062 (3)	0.037 (3)	0.106 (5)	−0.010 (2)	0.019 (3)	0.026 (3)
C9	0.046 (2)	0.024 (2)	0.077 (4)	−0.0073 (18)	−0.001 (2)	0.008 (2)
C10	0.077 (3)	0.022 (3)	0.099 (5)	−0.015 (2)	−0.001 (3)	−0.002 (3)
C11	0.076 (3)	0.028 (3)	0.087 (4)	−0.008 (2)	0.001 (3)	−0.019 (3)
C12	0.044 (2)	0.032 (2)	0.055 (3)	−0.0070 (17)	−0.001 (2)	−0.013 (2)
C13	0.057 (3)	0.047 (3)	0.053 (3)	−0.001 (2)	0.003 (2)	−0.026 (2)
C14	0.061 (3)	0.053 (3)	0.039 (3)	−0.015 (2)	0.014 (2)	−0.018 (2)
C15	0.038 (2)	0.035 (2)	0.039 (3)	−0.0058 (17)	0.0079 (18)	−0.0083 (19)
C16	0.0248 (18)	0.023 (2)	0.041 (2)	−0.0009 (14)	−0.0078 (16)	−0.0046 (17)
C17	0.0265 (19)	0.026 (2)	0.050 (3)	−0.0027 (15)	−0.0052 (17)	0.0024 (19)
N1	0.0261 (16)	0.0282 (17)	0.0347 (19)	−0.0050 (12)	−0.0029 (13)	−0.0002 (14)
N2	0.0312 (17)	0.0286 (18)	0.036 (2)	−0.0036 (13)	0.0020 (14)	0.0059 (15)
O5	0.0334 (14)	0.0221 (14)	0.0481 (18)	0.0020 (10)	−0.0158 (12)	−0.0086 (12)
O6	0.0308 (13)	0.0267 (14)	0.0294 (15)	−0.0083 (10)	−0.0074 (10)	−0.0034 (12)
C18	0.0214 (17)	0.030 (2)	0.025 (2)	−0.0053 (14)	0.0003 (14)	−0.0077 (16)
C19	0.0267 (19)	0.032 (2)	0.038 (2)	0.0019 (14)	−0.0105 (16)	−0.0133 (18)
C20	0.0301 (19)	0.028 (2)	0.035 (2)	−0.0071 (14)	−0.0072 (16)	−0.0081 (17)
C21	0.043 (2)	0.044 (3)	0.036 (2)	−0.0173 (18)	−0.0023 (18)	−0.010 (2)
C22	0.0267 (19)	0.031 (2)	0.033 (2)	−0.0054 (15)	−0.0039 (16)	−0.0077 (17)
O7	0.0433 (15)	0.0429 (17)	0.0338 (17)	−0.0169 (12)	0.0061 (12)	−0.0077 (13)
O8	0.0347 (14)	0.0405 (16)	0.0349 (16)	−0.0179 (11)	0.0054 (11)	−0.0118 (13)
N3	0.0293 (16)	0.0290 (18)	0.037 (2)	−0.0052 (12)	−0.0002 (14)	−0.0040 (15)
N4	0.0271 (16)	0.0334 (18)	0.0319 (19)	−0.0078 (12)	0.0002 (13)	−0.0030 (15)
C23	0.033 (2)	0.040 (2)	0.046 (3)	−0.0083 (17)	0.0017 (18)	−0.007 (2)
C24	0.032 (2)	0.049 (3)	0.064 (3)	−0.0100 (18)	0.009 (2)	−0.015 (2)
C25	0.026 (2)	0.054 (3)	0.063 (3)	−0.0017 (18)	0.000 (2)	−0.018 (2)
C26	0.032 (2)	0.043 (3)	0.043 (3)	0.0027 (17)	−0.0106 (18)	−0.018 (2)
C27	0.037 (2)	0.051 (3)	0.048 (3)	0.0100 (19)	−0.016 (2)	−0.003 (2)
C28	0.059 (3)	0.039 (3)	0.044 (3)	0.010 (2)	−0.019 (2)	0.002 (2)
C29	0.048 (2)	0.036 (2)	0.033 (2)	−0.0033 (18)	−0.0126 (19)	−0.0070 (19)
C30	0.069 (3)	0.033 (2)	0.037 (3)	−0.008 (2)	−0.011 (2)	0.004 (2)
C31	0.057 (3)	0.046 (3)	0.050 (3)	−0.017 (2)	0.004 (2)	0.007 (2)
C32	0.038 (2)	0.040 (3)	0.052 (3)	−0.0071 (18)	0.006 (2)	0.006 (2)
C33	0.035 (2)	0.029 (2)	0.029 (2)	−0.0054 (15)	−0.0077 (16)	−0.0068 (17)
C34	0.0282 (19)	0.035 (2)	0.030 (2)	−0.0003 (15)	−0.0102 (16)	−0.0093 (18)
O9	0.0385 (15)	0.0385 (17)	0.0366 (17)	0.0027 (12)	−0.0061 (12)	−0.0058 (13)
O10	0.0367 (14)	0.0544 (18)	0.0282 (15)	−0.0261 (12)	−0.0073 (11)	−0.0009 (13)
C35	0.0163 (17)	0.043 (2)	0.0202 (19)	−0.0097 (15)	0.0013 (14)	0.0051 (17)
C36	0.0257 (18)	0.036 (2)	0.028 (2)	−0.0121 (15)	−0.0047 (15)	0.0035 (17)
C37	0.0272 (19)	0.046 (2)	0.037 (2)	−0.0164 (16)	−0.0046 (16)	0.0134 (19)
C38	0.0220 (18)	0.034 (2)	0.031 (2)	−0.0068 (14)	0.0035 (15)	0.0038 (17)
C39	0.0189 (17)	0.029 (2)	0.027 (2)	−0.0054 (14)	−0.0026 (14)	0.0005 (16)
O11	0.0277 (13)	0.0332 (15)	0.0356 (16)	−0.0109 (10)	0.0061 (11)	−0.0043 (12)
O12	0.0242 (13)	0.0307 (14)	0.0343 (16)	−0.0075 (10)	0.0037 (11)	−0.0016 (12)

### Geometric parameters (Å, °)

**Table:**

Y1—O2^i^	2.314 (2)	C16—N1	1.358 (4)
Y1—O1^ii^	2.314 (2)	C16—C17	1.428 (5)
Y1—O6^iii^	2.315 (2)	C17—N2	1.366 (5)
Y1—O3	2.386 (2)	O5—C18	1.246 (4)
Y1—O5	2.441 (3)	O6—C18	1.270 (4)
Y1—O4	2.455 (3)	O6—Y1^iii^	2.315 (2)
Y1—O6	2.537 (2)	C18—C19	1.495 (5)
Y1—N2	2.538 (3)	C19—C20	1.505 (5)
Y1—N1	2.592 (3)	C19—H19A	0.9700
Y1—C5	2.779 (4)	C19—H19B	0.9700
Y1—C18	2.859 (4)	C20—C21	1.514 (5)
Y1—Y1^iii^	3.9205 (15)	C20—H20A	0.9700
Y2—O10^iv^	2.269 (3)	C20—H20B	0.9700
Y2—O12^v^	2.317 (2)	C21—C22	1.509 (5)
Y2—O11^i^	2.329 (2)	C21—H21A	0.9700
Y2—O7	2.375 (3)	C21—H21B	0.9700
Y2—O9	2.397 (3)	C22—O8	1.256 (4)
Y2—O8	2.413 (3)	C22—O7	1.260 (4)
Y2—N4	2.570 (3)	N3—C32	1.330 (4)
Y2—N3	2.649 (3)	N3—C33	1.367 (4)
Y2—C22	2.744 (4)	N4—C23	1.324 (4)
Y2—O10	2.823 (3)	N4—C34	1.352 (4)
Y2—C35	2.985 (4)	C23—C24	1.395 (5)
O1—C1	1.264 (4)	C23—H23A	0.9300
O1—Y1^ii^	2.314 (2)	C24—C25	1.351 (6)
O2—C1	1.255 (4)	C24—H24A	0.9300
O2—Y1^vi^	2.314 (2)	C25—C26	1.402 (6)
C1—C2	1.507 (4)	C25—H25A	0.9300
C2—C3	1.508 (5)	C26—C27	1.416 (6)
C2—H2A	0.9700	C26—C34	1.429 (5)
C2—H2B	0.9700	C27—C28	1.337 (6)
C3—C4	1.525 (4)	C27—H27A	0.9300
C3—H3A	0.9700	C28—C29	1.442 (5)
C3—H3B	0.9700	C28—H28A	0.9300
C4—C5	1.510 (5)	C29—C33	1.396 (5)
C4—H4A	0.9700	C29—C30	1.404 (6)
C4—H4B	0.9700	C30—C31	1.359 (6)
C5—O4	1.254 (4)	C30—H30A	0.9300
C5—O3	1.261 (4)	C31—C32	1.392 (5)
C6—N2	1.314 (5)	C31—H31A	0.9300
C6—C7	1.392 (6)	C32—H32A	0.9300
C6—H6A	0.9300	C33—C34	1.438 (5)
C7—C8	1.354 (7)	O9—C35	1.248 (4)
C7—H7A	0.9300	O10—C35	1.262 (4)
C8—C9	1.406 (6)	O10—Y2^iv^	2.269 (3)
C8—H8A	0.9300	C35—C36	1.502 (4)
C9—C17	1.411 (5)	C36—C37	1.526 (4)
C9—C10	1.427 (6)	C36—H36A	0.9700
C10—C11	1.343 (6)	C36—H36B	0.9700
C10—H10A	0.9300	C37—C38	1.523 (5)
C11—C12	1.424 (6)	C37—H37A	0.9700
C11—H11A	0.9300	C37—H37B	0.9700
C12—C13	1.402 (6)	C38—C39	1.522 (4)
C12—C16	1.416 (5)	C38—H38A	0.9700
C13—C14	1.362 (6)	C38—H38B	0.9700
C13—H13A	0.9300	C39—O11	1.253 (4)
C14—C15	1.400 (5)	C39—O12	1.261 (4)
C14—H14A	0.9300	O11—Y2^vi^	2.329 (2)
C15—N1	1.325 (4)	O12—Y2^v^	2.317 (2)
C15—H15A	0.9300		
			
O2^i^—Y1—O1^ii^	136.47 (8)	O4—C5—Y1	62.0 (2)
O2^i^—Y1—O6^iii^	77.41 (9)	O3—C5—Y1	58.93 (19)
O1^ii^—Y1—O6^iii^	73.73 (9)	C4—C5—Y1	178.2 (3)
O2^i^—Y1—O3	128.25 (9)	C5—O3—Y1	94.2 (2)
O1^ii^—Y1—O3	83.72 (9)	C5—O4—Y1	91.1 (2)
O6^iii^—Y1—O3	89.75 (9)	N2—C6—C7	123.8 (4)
O2^i^—Y1—O5	80.79 (9)	N2—C6—H6A	118.1
O1^ii^—Y1—O5	89.26 (9)	C7—C6—H6A	118.1
O6^iii^—Y1—O5	124.28 (8)	C8—C7—C6	119.0 (4)
O3—Y1—O5	141.65 (9)	C8—C7—H7A	120.5
O2^i^—Y1—O4	74.53 (8)	C6—C7—H7A	120.5
O1^ii^—Y1—O4	128.63 (9)	C7—C8—C9	120.1 (4)
O6^iii^—Y1—O4	78.31 (9)	C7—C8—H8A	120.0
O3—Y1—O4	53.73 (8)	C9—C8—H8A	120.0
O5—Y1—O4	141.72 (9)	C8—C9—C17	117.0 (4)
O2^i^—Y1—O6	68.63 (8)	C8—C9—C10	124.1 (4)
O1^ii^—Y1—O6	71.91 (8)	C17—C9—C10	118.8 (4)
O6^iii^—Y1—O6	72.29 (10)	C11—C10—C9	121.6 (4)
O3—Y1—O6	152.82 (8)	C11—C10—H10A	119.2
O5—Y1—O6	52.02 (8)	C9—C10—H10A	119.2
O4—Y1—O6	136.72 (8)	C10—C11—C12	120.9 (4)
O2^i^—Y1—N2	77.91 (9)	C10—C11—H11A	119.6
O1^ii^—Y1—N2	139.47 (9)	C12—C11—H11A	119.6
O6^iii^—Y1—N2	145.78 (10)	C13—C12—C16	117.0 (4)
O3—Y1—N2	87.22 (10)	C13—C12—C11	123.2 (4)
O5—Y1—N2	74.03 (10)	C16—C12—C11	119.7 (4)
O4—Y1—N2	72.51 (10)	C14—C13—C12	120.3 (4)
O6—Y1—N2	119.13 (9)	C14—C13—H13A	119.9
O2^i^—Y1—N1	136.02 (9)	C12—C13—H13A	119.9
O1^ii^—Y1—N1	75.86 (9)	C13—C14—C15	118.6 (4)
O6^iii^—Y1—N1	146.27 (8)	C13—C14—H14A	120.7
O3—Y1—N1	72.67 (9)	C15—C14—H14A	120.7
O5—Y1—N1	69.05 (9)	N1—C15—C14	123.6 (4)
O4—Y1—N1	110.76 (9)	N1—C15—H15A	118.2
O6—Y1—N1	111.37 (9)	C14—C15—H15A	118.2
N2—Y1—N1	63.74 (10)	N1—C16—C12	122.6 (4)
O2^i^—Y1—C5	101.34 (10)	N1—C16—C17	118.5 (3)
O1^ii^—Y1—C5	106.81 (10)	C12—C16—C17	118.9 (3)
O6^iii^—Y1—C5	83.31 (10)	N2—C17—C9	122.3 (4)
O3—Y1—C5	26.92 (9)	N2—C17—C16	117.5 (3)
O5—Y1—C5	151.60 (9)	C9—C17—C16	120.1 (4)
O4—Y1—C5	26.82 (9)	C15—N1—C16	117.8 (3)
O6—Y1—C5	155.01 (9)	C15—N1—Y1	123.8 (2)
N2—Y1—C5	78.72 (11)	C16—N1—Y1	118.3 (2)
N1—Y1—C5	91.86 (10)	C6—N2—C17	117.9 (3)
O2^i^—Y1—C18	72.41 (9)	C6—N2—Y1	121.5 (3)
O1^ii^—Y1—C18	80.43 (9)	C17—N2—Y1	120.1 (2)
O6^iii^—Y1—C18	98.61 (10)	C18—O5—Y1	96.2 (2)
O3—Y1—C18	159.19 (9)	C18—O6—Y1^iii^	160.8 (2)
O5—Y1—C18	25.67 (8)	C18—O6—Y1	91.13 (19)
O4—Y1—C18	146.64 (8)	Y1^iii^—O6—Y1	107.71 (10)
O6—Y1—C18	26.36 (8)	O5—C18—O6	120.5 (3)
N2—Y1—C18	96.11 (11)	O5—C18—C19	121.3 (3)
N1—Y1—C18	90.35 (10)	O6—C18—C19	118.1 (3)
C5—Y1—C18	172.75 (10)	O5—C18—Y1	58.08 (18)
O2^i^—Y1—Y1^iii^	68.62 (6)	O6—C18—Y1	62.51 (17)
O1^ii^—Y1—Y1^iii^	68.51 (6)	C19—C18—Y1	175.0 (2)
O6^iii^—Y1—Y1^iii^	38.06 (6)	C18—C19—C20	115.8 (3)
O3—Y1—Y1^iii^	124.96 (7)	C18—C19—H19A	108.3
O5—Y1—Y1^iii^	86.24 (6)	C20—C19—H19A	108.3
O4—Y1—Y1^iii^	110.58 (7)	C18—C19—H19B	108.3
O6—Y1—Y1^iii^	34.23 (6)	C20—C19—H19B	108.3
N2—Y1—Y1^iii^	143.45 (6)	H19A—C19—H19B	107.4
N1—Y1—Y1^iii^	136.64 (7)	C19—C20—C21	113.9 (3)
C5—Y1—Y1^iii^	121.18 (8)	C19—C20—H20A	108.8
C18—Y1—Y1^iii^	60.56 (8)	C21—C20—H20A	108.8
O10^iv^—Y2—O12^v^	77.40 (9)	C19—C20—H20B	108.8
O10^iv^—Y2—O11^i^	75.51 (9)	C21—C20—H20B	108.8
O12^v^—Y2—O11^i^	133.77 (9)	H20A—C20—H20B	107.7
O10^iv^—Y2—O7	148.71 (9)	C22—C21—C20	114.2 (3)
O12^v^—Y2—O7	88.66 (9)	C22—C21—H21A	108.7
O11^i^—Y2—O7	131.21 (9)	C20—C21—H21A	108.7
O10^iv^—Y2—O9	124.70 (9)	C22—C21—H21B	108.7
O12^v^—Y2—O9	76.41 (9)	C20—C21—H21B	108.7
O11^i^—Y2—O9	89.28 (9)	H21A—C21—H21B	107.6
O7—Y2—O9	77.41 (10)	O8—C22—O7	121.1 (4)
O10^iv^—Y2—O8	146.55 (8)	O8—C22—C21	119.6 (3)
O12^v^—Y2—O8	135.99 (9)	O7—C22—C21	119.3 (3)
O11^i^—Y2—O8	76.75 (9)	O8—C22—Y2	61.55 (19)
O7—Y2—O8	54.46 (8)	O7—C22—Y2	59.85 (19)
O9—Y2—O8	72.94 (9)	C21—C22—Y2	174.3 (3)
O10^iv^—Y2—N4	84.42 (10)	C22—O7—Y2	92.8 (2)
O12^v^—Y2—N4	135.98 (9)	C22—O8—Y2	91.2 (2)
O11^i^—Y2—N4	77.18 (9)	C32—N3—C33	116.5 (3)
O7—Y2—N4	86.64 (10)	C32—N3—Y2	123.9 (2)
O9—Y2—N4	143.92 (10)	C33—N3—Y2	119.5 (2)
O8—Y2—N4	71.46 (10)	C23—N4—C34	117.6 (3)
O10^iv^—Y2—N3	77.12 (10)	C23—N4—Y2	120.4 (2)
O12^v^—Y2—N3	74.34 (9)	C34—N4—Y2	122.0 (2)
O11^i^—Y2—N3	132.89 (9)	N4—C23—C24	124.2 (4)
O7—Y2—N3	72.15 (10)	N4—C23—H23A	117.9
O9—Y2—N3	137.77 (10)	C24—C23—H23A	117.9
O8—Y2—N3	109.74 (9)	C25—C24—C23	118.5 (4)
N4—Y2—N3	62.57 (9)	C25—C24—H24A	120.7
O10^iv^—Y2—C22	163.37 (10)	C23—C24—H24A	120.7
O12^v^—Y2—C22	112.29 (10)	C24—C25—C26	120.5 (4)
O11^i^—Y2—C22	103.94 (10)	C24—C25—H25A	119.8
O7—Y2—C22	27.30 (9)	C26—C25—H25A	119.8
O9—Y2—C22	71.66 (10)	C25—C26—C27	124.3 (4)
O8—Y2—C22	27.24 (9)	C25—C26—C34	116.9 (4)
N4—Y2—C22	79.32 (10)	C27—C26—C34	118.8 (4)
N3—Y2—C22	92.18 (11)	C28—C27—C26	121.9 (4)
O10^iv^—Y2—O10	76.57 (10)	C28—C27—H27A	119.0
O12^v^—Y2—O10	68.15 (8)	C26—C27—H27A	119.0
O11^i^—Y2—O10	69.54 (8)	C27—C28—C29	121.2 (4)
O7—Y2—O10	124.08 (8)	C27—C28—H28A	119.4
O9—Y2—O10	48.56 (9)	C29—C28—H28A	119.4
O8—Y2—O10	110.43 (9)	C33—C29—C30	118.2 (4)
N4—Y2—O10	144.79 (8)	C33—C29—C28	118.9 (4)
N3—Y2—O10	137.84 (8)	C30—C29—C28	122.9 (4)
C22—Y2—O10	119.18 (9)	C31—C30—C29	119.2 (4)
O10^iv^—Y2—C35	101.03 (11)	C31—C30—H30A	120.4
O12^v^—Y2—C35	69.16 (9)	C29—C30—H30A	120.4
O11^i^—Y2—C35	80.16 (9)	C30—C31—C32	118.9 (4)
O7—Y2—C35	99.85 (10)	C30—C31—H31A	120.6
O9—Y2—C35	23.74 (10)	C32—C31—H31A	120.6
O8—Y2—C35	92.24 (10)	N3—C32—C31	124.4 (4)
N4—Y2—C35	154.55 (9)	N3—C32—H32A	117.8
N3—Y2—C35	142.85 (9)	C31—C32—H32A	117.8
C22—Y2—C35	95.17 (11)	N3—C33—C29	122.8 (3)
O10—Y2—C35	24.90 (9)	N3—C33—C34	117.1 (3)
O10^iv^—Y2—Y2^iv^	43.19 (7)	C29—C33—C34	120.1 (3)
O12^v^—Y2—Y2^iv^	67.34 (7)	N4—C34—C26	122.2 (3)
O11^i^—Y2—Y2^iv^	67.20 (6)	N4—C34—C33	118.7 (3)
O7—Y2—Y2^iv^	151.36 (6)	C26—C34—C33	119.1 (4)
O9—Y2—Y2^iv^	81.74 (7)	C35—O9—Y2	105.6 (2)
O8—Y2—Y2^iv^	135.85 (7)	C35—O10—Y2^iv^	166.4 (2)
N4—Y2—Y2^iv^	121.33 (8)	C35—O10—Y2	84.7 (2)
N3—Y2—Y2^iv^	113.30 (7)	Y2^iv^—O10—Y2	103.43 (10)
C22—Y2—Y2^iv^	152.26 (8)	O9—C35—O10	120.7 (3)
O10—Y2—Y2^iv^	33.38 (6)	O9—C35—C36	120.1 (3)
C35—Y2—Y2^iv^	57.99 (8)	O10—C35—C36	119.2 (3)
C1—O1—Y1^ii^	132.1 (2)	O9—C35—Y2	50.68 (18)
C1—O2—Y1^vi^	135.6 (2)	O10—C35—Y2	70.3 (2)
O2—C1—O1	126.3 (3)	C36—C35—Y2	167.8 (3)
O2—C1—C2	117.9 (3)	C35—C36—C37	109.4 (3)
O1—C1—C2	115.8 (3)	C35—C36—H36A	109.8
C1—C2—C3	116.5 (3)	C37—C36—H36A	109.8
C1—C2—H2A	108.2	C35—C36—H36B	109.8
C3—C2—H2A	108.2	C37—C36—H36B	109.8
C1—C2—H2B	108.2	H36A—C36—H36B	108.2
C3—C2—H2B	108.2	C38—C37—C36	115.1 (3)
H2A—C2—H2B	107.3	C38—C37—H37A	108.5
C2—C3—C4	111.6 (3)	C36—C37—H37A	108.5
C2—C3—H3A	109.3	C38—C37—H37B	108.5
C4—C3—H3A	109.3	C36—C37—H37B	108.5
C2—C3—H3B	109.3	H37A—C37—H37B	107.5
C4—C3—H3B	109.3	C39—C38—C37	111.0 (3)
H3A—C3—H3B	108.0	C39—C38—H38A	109.4
C5—C4—C3	115.7 (3)	C37—C38—H38A	109.4
C5—C4—H4A	108.4	C39—C38—H38B	109.4
C3—C4—H4A	108.4	C37—C38—H38B	109.4
C5—C4—H4B	108.4	H38A—C38—H38B	108.0
C3—C4—H4B	108.4	O11—C39—O12	126.0 (3)
H4A—C4—H4B	107.4	O11—C39—C38	117.4 (3)
O4—C5—O3	121.0 (3)	O12—C39—C38	116.6 (3)
O4—C5—C4	118.8 (3)	C39—O11—Y2^vi^	138.4 (2)
O3—C5—C4	120.2 (3)	C39—O12—Y2^v^	138.6 (2)

Symmetry codes: (i) *x*+1, *y*, *z*; (ii) −*x*+1, −*y*, −*z*+1; (iii) −*x*+2, −*y*, −*z*+1; (iv) −*x*+2, −*y*+1, −*z*+2; (v) −*x*+1, −*y*+1, −*z*+2; (vi) *x*−1, *y*, *z*.
